# Recent advances in human iPSC-derived models of the blood–brain barrier

**DOI:** 10.1186/s12987-020-00191-7

**Published:** 2020-04-22

**Authors:** Michael J. Workman, Clive N. Svendsen

**Affiliations:** Board of Governors Regenerative Medicine Institute, Cedars-Sinai Medical Center, Los Angeles, CA USA

**Keywords:** Blood–brain barrier, Induced pluripotent stem cells, Human iPSC, Disease modeling, Brain microvascular endothelial cells, Organ-chip systems

## Abstract

The blood–brain barrier (BBB) is a critical component of the central nervous system that protects neurons and other cells of the brain parenchyma from potentially harmful substances found in peripheral circulation. Gaining a thorough understanding of the development and function of the human BBB has been hindered by a lack of relevant models given significant species differences and limited access to in vivo tissue. However, advances in induced pluripotent stem cell (iPSC) and organ-chip technologies now allow us to improve our knowledge of the human BBB in both health and disease. This review focuses on the recent progress in modeling the BBB in vitro using human iPSCs.

## Introduction

The blood–brain barrier (BBB) is formed by specialized brain microvascular endothelial cells (BMECs) and other supporting cells of the neurovascular unit (NVU) including pericytes, astrocytes, and neurons. These cells form an extremely selective barrier that prevents potentially harmful compounds in the blood from diffusing into the central nervous system (CNS), thus protecting neurons from blood-borne neurotoxins and microbial infections. The BBB forms early in development as cells from the perineural vascular plexus invade the neuroectoderm to vascularize the CNS. Additional signaling cues from neurons and other cells of the CNS further specify BMECs to become the highly specialized brain endothelium [1]. Entry of molecules into the CNS is tightly controlled through various transporters expressed by BMECs, which presents many challenges when developing drugs and other therapeutics intended to target the brain. Species-specific differences in the type and expression level of a number of these transporters [2–6] limit the utility of animal models in preclinical studies. Isolation of primary human BMECs [7] and generation of immortalized human BMEC cell lines [8, 9] have permitted modeling of the human BBB in vitro, but limitations such as access to postmortem tissue or lack of sufficient barrier properties of these cells have hindered their potential as accurate models. To overcome these shortcomings, researchers have turned to induced pluripotent stem cells (iPSCs) as a renewable source of BMECs for in vitro BBB modeling. iPSCs can be generated from adult somatic cells to produce a theoretically unlimited number of cells carrying the donor’s genetic makeup that have the ability to differentiate into any cell of the body [10, 11].

In the past decade, advances in human iPSC technology have enabled the generation of BMECs from iPSCs [12]. These cells display many key characteristics of bona fide BMECs, including proper organization of tight junctions and appropriate expression of nutrient and efflux transporters. Furthermore, these cells form an effective barrier measured by trans-endothelial electrical resistance (TEER) and have drug permeabilities that highly correlate with in vivo measurements [12]. This and several other early BBB models were based on transwell systems comprised of cells seeded on permeable inserts that divide a cell culture well into upper and lower compartments, allowing for both apical and basolateral media delivery and co-culture of other cell types. Unfortunately, these static platforms lack the critical component of media flow and sheer stress that BMECs are constantly exposed to in vivo.

**Fig. 1:**
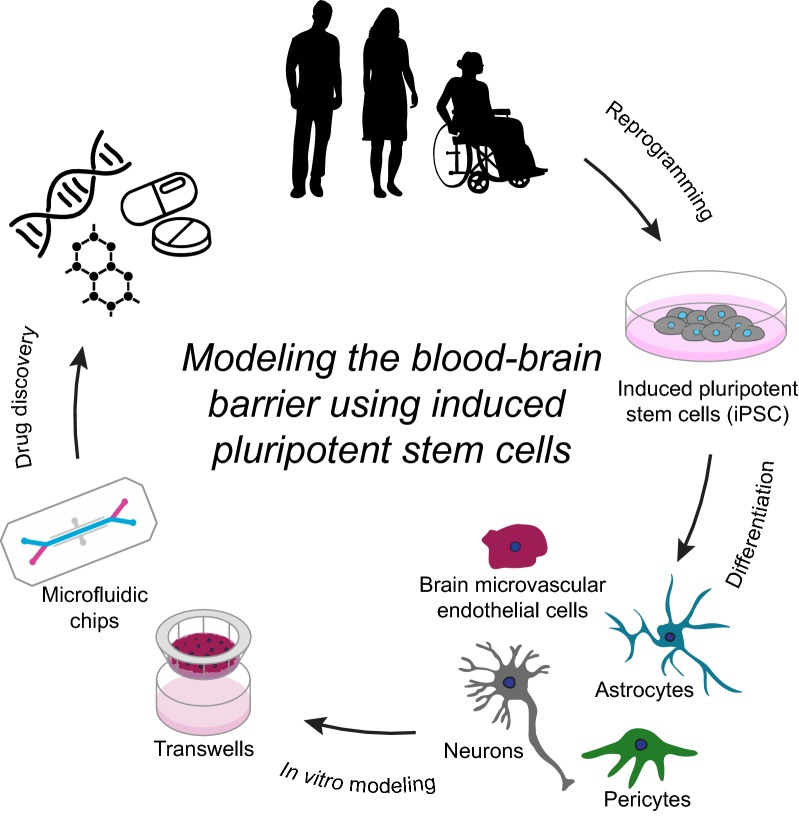
Overview of modeling the blood–brain barrier using induced pluripotent stem cells

Recent advancements in organ-chip technology have overcome this limitation and significant enhancements in the differentiation protocol have followed in the years since the seminal work of Lippmann et al. [12]. iPSC-derived BMECs (iBMECs) have substantially advanced in vitro modeling of the human BBB, thereby increasing our knowledge of human BBB development and function as well as facilitating CNS drug discovery (Fig. 1). In this review we focus on the most recent technological advances in BBB modeling using human iPSCs and the innovative ways iBMECs are being used to predict drug permeabilities and gain new insights into human development and neurological disease (Table 1).

**Table 1:** Comparison of major culture platforms used for in vitro BBB models 
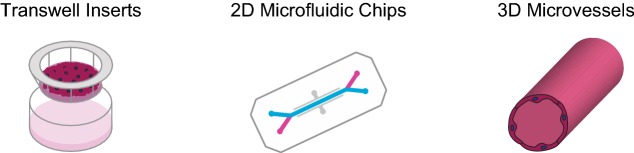

Advantages	Highly scalable; easily measure TEER; relatively simple model for drug permeability studies; allows for investigation of paracrine signaling	Replicates in vivo physiological forces of flow and stretch; allows cell–cell contacts; mimics vasculature with microfluidic channels	Geometry mimics in vivo vessels; replicates physiological shear stress and cell–matrix interactions
Challenges	Static culture conditions; lack of cell–cell contacts in co-culture	Limited scalability; expensive; requires specialized expertise for manufacturing; drug absorption by materials such as PDMS	Low throughput; difficult to measure TEER values and drug permeabilities; challenges with long-term stability
Response to shear stress		DeStefano [60]; Wang [64]; Vatine [31]	Faley [63]; Linville [67]
NVU cell–cell interactions	Lippmann [13]; Appelt-Menzel [24]; Canfield [26, 27]; Hollman [17]; Delsing [25, 28]; Mantle [45]; Stebbins [29]	Motallebnejad [66]; Park [19]; Vatine [31]; Jagadeesan [58]	Campisi [57]; Jamieson [32]
Drug permeability and drug delivery	Lippmann [12]; Mantle [73]; Appelt-Menzel [24]; Delsing [25]; Ribecco-Lutkiewicz [30]; Le Roux [74]; Li [71]; Ohshima [69]	Wang [64]; Park [19]; Vatine [31]	Linville [67]; Lee [65]
Neurological disease modeling	Qosa [46]; Lim [39]; Vatine [38]; Lee [41]; Al-Ahmad [72]; Katt [40]; Mantle [85]; Mohamed [47]; Page [48]	Motallebnejad [66]; Vatine [31]	Shin [43]
Infectious disease modeling	Kim [49, 50]; Alimonti [53]; Patel [52]; Martins Gomes [51]		

Key recent iPSC-derived BBB studies utilizing each platform are listed according to main area of research

## Improvements in BMEC differentiation methods

**Fig. 2:**
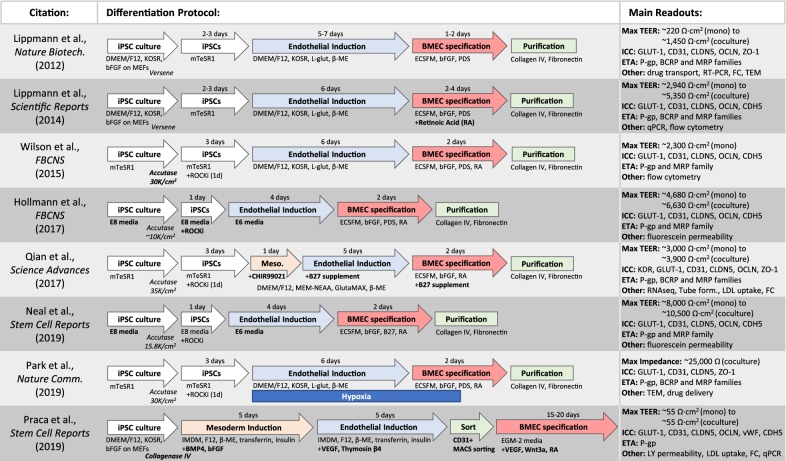
Schematic of differentiation protocols for deriving brain microvascular endothelial cells from induced pluripotent stem cells and main assay readouts for assessing BMEC phenotype. Main advancements from previous protocols are bolded. *bFGF* basic fibroblast growth factor, *MEF* mouse embryonic fibroblast, *KOSR* knockout serum replacement, *l-glut*l-glutamine, *β-ME* β-mercaptoethanol, *ECSFM* endothelial cell serum free media, *PDS* platelet-poor plasma derived serum, *VEGF* vascular endothelial growth factor, *TEER* transendothelial electrical resistance, *ICC* immunocytochemistry, *ETA* efflux transporter activity, *FC* flow cytometry, *TEM* transmission electron microscopy, *RT-PCR* reverse transcription polymerase chain reaction, *qPCR* quantitative polymerase chain reaction

With an increased understanding of BBB development based on the molecular signaling events that occur during embryogenesis, considerable improvements in differentiating BMECs from iPSCs have been made in recent years (Fig. 2). Early protocols sought to mimic the brain microenvironment utilizing a strategy of endothelial and neural co-differentiation in unconditioned media, followed by a BMEC specification and expansion stage in endothelial cell media [12]. Following differentiation, iBMECs are then selectively purified on a mixture of collagen and fibronectin. The addition of retinoic acid (RA) during the BMEC specification stage was a major advancement that substantially increases both the differentiation efficiency and barrier properties acquired by iBMECs [13, 14]. Until recently, relatively little was known about the mechanism by which RA treatment leads to increases in adherens and tight junction expression and the subsequent enhanced barrier properties. However, studies aimed at examining the effects of RA have revealed how activation of specific RA receptors and retinoid X receptors in iBMECs using selective small molecule agonists can mimic the effects of RA treatment in an overlapping and synergistic manner [15]. Furthermore, previously unappreciated paracrine signals from RA-stimulated neural cells that co-differentiate with iBMECs also contribute to the enhancement of barrier properties induced by RA [15]. Further improvements including optimizing initial iPSC seeding density [16] and accelerating the differentiation time [17] have continued to improve the generation of iBMECs from iPSCs. More recent efforts to perfect iBMEC differentiation have focused on incorporating signaling cues that mirror in vivo vasculogenesis. For example, adding CHIR99021, a small molecule Wnt/β-catenin agonist, early in the differentiation procedure promotes an intermediate mesoderm stage and subsequent endothelial cell specification that more accurately mimics the developmental trajectory of BMECs in vivo [18]. Along similar lines, by simulating the low oxygen environment BMECs are exposed to during development, incorporation of hypoxia during differentiation significantly enhances barrier properties including increased TEER and expression of efflux transporters that approach in vivo levels [19]. Importantly, the protocols mentioned above continue to benchmark differentiated cells using TEER, efflux transporter activity (ETA), and expression of essential BBB junctional proteins (Claudin-5, ZO-1, Occludin, VE-Cadherin), transporters (P-gp, GLUT1), and other factors (PECAM-1, VEGFR2, vWF). Additional recent improvements in the differentiation of iBMECs include the use of fully defined media [20], sorting strategies to increase iBMEC purity [21], and effective methods for the cryopreservation of differentiated cells [22, 23], which collectively are improving the reproducibility and scalability of iBMECs for laboratory and potential clinical use.

## BBB development and neurological disease

Human iPSC-derived BBB models have been used to understand both BBB development and the impact of other cells of the NVU on barrier formation. Several groups have found that co-culture of iBMECs with primary astrocytes, pericytes, and neural cells significantly enhances barrier formation as measured by TEER and permeability to various molecules [13, 24, 25]. More recent work has focused on differentiating iPSCs into the additional cells of the NVU and combining these with iBMECs for fully iPSC-derived BBB models. Similar to results with primary cells, co-culture of iBMECs with iPSC-derived cells of the NVU can also raise TEER values and improve barrier function [26–31], however these additional cells are not required for iBMECs to achieve physiological TEER levels and may only improve barrier properties under suboptimal starting conditions or stress [32]. These studies have initiated a personalized approach to BBB modeling, which will likely provide new insights into genetic-based neurological diseases that may involve cell–cell interactions of the NVU. The focus on these cellular interactions has largely been on how the cells of the NVU affect BMECs and barrier formation during development, however other studies have highlighted how iBMECs enhance neuronal maturation and function. When co-cultured, iBMECs promote an increase in spontaneous activity of iPSC-derived motor neurons and induce gene signatures indicative of more mature neuronal cells [33].

Barrier breakdown and dysfunction has been observed in nearly all major neurodegenerative diseases and likely contributes to the initiation and progression of pathology in many neurological disorders [34–37]. The application of iPSC-derived BBB models has contributed to the understanding of BBB dysfunction and has established the ability to study disease mechanisms in a personalized manner. This approach also offers the opportunity to investigate the earliest stages of BBB breakdown associated with disease, which can be difficult to ascertain from postmortem tissue. Recent work from our lab and others has focused on modeling monogenic neurological disorders using iPSC-derived BBB models, which have provided new insights into disease mechanisms. For example, we determined that Allan-Herndon-Dudley syndrome, caused by mutations in *SLC16A2* encoding a thyroid hormone (TH) transporter, involves inadequate transport of TH across the BBB rather than an inability of neural cells to utilize TH [31, 38]. Human iPSC-derived BBB models have also been used to show that other monogenic neurological diseases such as Huntington’s disease (HD) [31, 39, 40] and cerebral adrenoleukodystrophy [41] both display barrier defects in iBMECs differentiated from patient iPSCs, suggesting that BBB breakdown is a contributing factor to disease. While the signaling pathways associated with BBB breakdown have been difficult to elucidate, recent work with iPSC-derived BBB models are beginning to uncover specific molecules, such as hyaluronan, that can negatively impact barrier integrity through interaction with the CD44 receptor [42]. BBB dysfunction has also been observed in several iPSC-derived BBB models of the most common neurodegenerative diseases. Using iPSCs from patients with familial forms of neurodegenerative disease, including Alzheimer’s disease (AD), Parkinson’s disease (PD), and amyotrophic lateral sclerosis (ALS), Katt and colleagues [40] showed that these patient-derived iBMECs had various forms of BBB impairment, such as a decrease in TEER and rhodamine 123 efflux ratio or an increase in Lucifer yellow and d-glucose permeability, compared to healthy controls. Familial AD mutations also cause a reduction in the expression of tight junction proteins and are associated with increases in BBB permeability and the deposition of β-amyloid (Aβ) on the surface of iBMECs [43]. Furthermore, variants in the *APOE* gene, representing one of the most well-known risk factors for AD, result in increased production of proinflammatory cytokines and Aβ by iBMECs [44]. These issues can potentially be alleviated by co-culturing iBMECs with healthy control astrocytes, which can mitigate barrier dysfunction associated with exposing iBMECs to proinflammatory cytokines, such as tumor necrosis factor alpha (TNFα) and interleukin 6 (IL-6) [45]. Interestingly, in ALS iPSC-derived astrocytes from SOD1 and sporadic patients cause an upregulation of P-gp in co-cultured iBMECs [46, 47]. The upregulation of this efflux transporter may limit the delivery of therapeutics to the CNS but could also serve to protect the brain if there is any disease-associated loss of barrier integrity. In addition to studies of neurodegenerative disease, iBMECs have also been used in in vitro models of stroke and remarkably, recapitulate the hallmark BBB disruption associated with brain ischemia when subjected to oxygen–glucose deprivation [48]. Taken together, these results indicate that BBB dysfunction—a common feature of many neurological disorders—can be modeled using iPSC technology and that neuronal loss associated with disease is likely not entirely cell autonomous.

## Infectious disease

Several infectious diseases affecting the CNS involve BMECs since pathogens must pass through the BBB to infect the CNS. Many of these infectious agents are human-specific and have been difficult to study with animal models. Using human iBMECs, significant advances have been made in understanding the host response to several bacteria and viruses. For example, bacterial meningitis is a life-threatening infection caused by a variety of bacteria that enter the CNS through a compromised BBB, leading to inflammation of the meninges. Two meningitis-causing bacteria have been studied using iBMECs: *Streptococcus agalactiae* and *Neisseria meningitidis*. Interestingly, iBMECs respond to *S. agalactiae* infection by upregulating cytokines and chemokines such as IL-8 and *CXCL1* that are involved in neutrophil recruitment, mimicking in vivo response to infection [49]. Furthermore, *S. agalactiae* inhibits the key BBB efflux transporter *P*-glycoprotein, a previously unknown effect of infection discovered using iBMEC models [50]. Similar results have been seen in iBMECs infected with *N. meningitidis* in which bacterial challenge results in upregulation of proinflammatory cytokines and disruption of tight junctions [51]. Beyond bacterial infection, iBMECs are also being used to investigate fungal and viral infections of the CNS. Mechanisms by which these pathogens enter the brain are poorly understood, but studies using iBMECs have elucidated ways that viruses and fungi breach the BBB. For example, gliotoxin secreted by *Aspergillus fumigatus* decreases TEER and increases BBB permeability, likely permitting fungal invasion [52]. Surprisingly, barrier disruption in this model occurred independent of changes to tight junctions and rather by a previously unknown mechanism of impairment in cell–matrix interactions [52]. iBMECs have also been used for revealing how Zika virus crosses the BBB through paracellular diapedesis to infect the CNS without compromising BBB integrity [53]. These studies highlight that iBMECs can be used to elucidate how human-specific pathogens traverse the BBB to colonize the CNS and could guide the development of new therapeutics to combat infection.

## iPSC-derived microfluidic chip models of the BBB

One of the most noteworthy recent advances in iPSC-derived models of the BBB has come in the generation of bioengineered microfluidic organ-chip-based models [54–56]. These microphysiological systems typically incorporate iBMECs into hydrogels [57] or polydimethylsiloxane (PDMS)-based devices [58] that attempt to recreate the anatomical, physiological and mechanical forces that cells experience in vivo. Much of this work has been reviewed previously [59], and thus, we focus on several recent advances that have extended progress in this area. One advantage of using microfluidic devices is the ability to apply fluid flow. Utilizing this capability, several groups have lined microfluidic devices with iBMECs to study the cellular response to flow-induced shear stress. Using a PDMS-based platform with shear rates up to 12 dyne/cm^2^, investigators determined that iBMECs do not elongate or align with the direction of fluid flow in response to shear stress, a phenotype unique to endothelial cells of the brain [60]. Expanding on these observations, subsequent studies showed that while iBMECs do not change their morphology in response to shear stress, they do respond at the transcriptional level in a force-dependent manner [31]. Mirroring in vivo responses, addition of the proinflammatory cytokine TNFα to the fluid flow causes an upregulation of adhesion molecules ICAM-1 and VCAM-1 on the surface of iBMECs and results in an increased adherence of leukocytes perfused through the organ-chip, demonstrating the ability to model important inflammatory processes in vitro [61]. Many of these organ-chip platforms are based on 2-dimensional models of the BBB, which are a single vascular channel that do not replicate the geometry or complex network of vessels formed in vivo. Using a combination of iBMECs and other cells of the NVU embedded in a fibrin gel, researchers have created a perfusable 3-dimensional microvascular network organ-chip [57]. One of the challenges has been maintaining effective long-term barrier properties with iBMECs. Several groups have made progress in this area by exposing iBMECs to hypoxia [19], modifying extracellular matrix composition and stiffness [62], or by optimizing hydrogel scaffolds and fluid flow parameters that allow for maintenance of barrier properties for up to 3 weeks [63]. Many of the BBB-chip platforms developed and optimized recently are now being used to investigate drug permeabilities [19, 31, 64, 65], neurodegenerative disease [31, 43, 66], and other functional aspects of the BBB [33, 60, 61, 67].

## Drug transport and delivery

Designing and testing BBB-permeable drugs represents a huge burden for CNS drug development. The vast majority of compounds—approximately 100% of large molecules and more than 98% of small molecules—are excluded from the CNS by the BBB through the physical barrier or by efflux pumps expressed by BMECs [68]. Due to the species-specific differences in transporter and efflux pump expression, human iPSC-based BBB models are an attractive platform to test drug permeability. These models more accurately predict human BBB permeability compared to non-human BBB models [69] and hold great promise in providing a high-throughput platform for predicting human CNS drug permeabilities and circumventing the need for animal-based testing [70]. Early iPSC-derived BBB models highlighted the ability of iBMECs to correlate well with in vivo drug permeability using transwell systems [12, 71] and subsequent studies have expanded permeability testing to microfluidic platforms under fluid flow that more closely mimic in vivo conditions [19, 31, 64]. Importantly, iBMECs express many of the necessary efflux pumps and transporters [18, 31, 39] and have successfully been used to investigate general drug transport as well as specific transporter–drug interactions such as LAT1 with gabapentin [72]. Furthermore, iBMECs can be co-cultured with other cells of the NVU that can potentially alter drug permeabilities through changes in barrier properties or transporter expression [24], and hence should be considered when designing drug screening platforms. However, it is worth noting that permeability for candidate large and small molecules does not change above TEER thresholds of 500 and 900 Ω cm^2^, respectively [73], suggesting that complex co-culture models may not be necessary for accurately modeling permeability. Despite these advances in drug permeability testing using iPSC-derived BBB models, limited in vivo human permeability data is available to benchmark in vitro BBB models. Recent work has begun to address this issue by measuring in vitro permeability of positron emission tomography (PET) radioligands, for which in vivo human BBB permeability values are known from clinical PET imaging [74]. Remarkably, iPSC-derived BBB models show highly significant correlation to in vivo values for the 8 radioligands tested. Interestingly, when Le Roux and colleagues [74] tested a suite of other drugs in their radioligand-validated model, they generated relative permeabilities that could not have been predicted based on the physicochemical properties of the drugs alone. In addition to permeability testing of small molecules, iPSC-derived BBB models are also being used to test permeability of new classes of CNS drugs such as peptides and antibodies. For example, attaching an Angiopep-2 peptide to fluorescent nanoparticles can increase their BBB permeability by 3.5-fold [19] and a comparable strategy could be used to increase CNS delivery of larger molecule therapeutics. Similarly, iPSC-derived BBB models are being used to evaluate receptor mediated transcytosis-targeting antibodies to enhance drug delivery [30], also known as molecular Trojan horses [75]. The species-specific differences in transporter expression highlighted earlier [2–6] underscores the importance of using human-based models for testing these types of novel delivery mechanisms. Lastly, other alternative drug delivery strategies being explored using iPSC-derived BBB models are polymer nanoparticles [65] and perfusion of hyperosmolar agents like mannitol to temporarily open the BBB and permit the diffusion of non-permeable therapeutics into the CNS [19, 61, 67]. The development of iPSC-derived BBB models has significantly enhanced the ability to perform human-relevant in vitro drug screens and will likely continue to aid in the discovery and development of new therapeutics and CNS drug delivery methods.

## Challenges and future directions

**Fig. 3:**
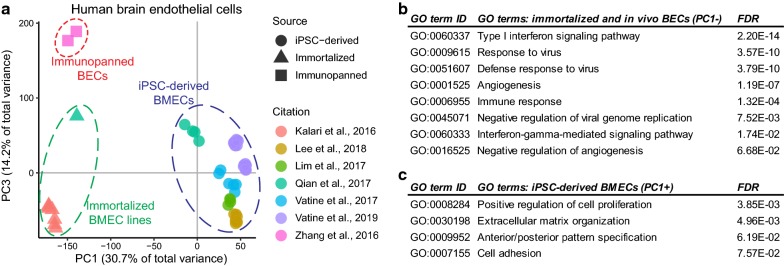
**a** Principal component analysis of published RNA-sequencing data comparing transcriptomes of human iPSC-derived brain microvascular endothelial cells (iBMECs), immortalized BMEC cell lines, and immunopanned brain endothelial cells (BECs) from post-mortem samples. The first principal component (PC1), representing the largest proportion of explained variance, separates iPSC-derived from in vivo-sourced brain endothelial cells. **b** Gene ontology (GO) enrichment using the top 400 genes driving the separation of samples along PC1 reveal that pathways associated with immune signaling and angiogenesis are upregulated in immunopanned BECs and immortalized cell lines. **c** Conversely, iPSC-derived BMECs show upregulation of terms associated with cell proliferation, patterning, and extracellular matrix interaction

Human iPSC-derived BMECs have had a significant impact on improving our understanding of human BBB development and disease. These cells recapitulate many morphological, functional, and molecular features of in vivo BMECs and have proven useful for modeling the human BBB under various conditions ranging from normal homeostasis to neurological disease and infection. However, like most iPSC-derived cells, iBMECs do not fully recapitulate all aspects of their in vivo counterparts. For instance, transcriptomic analyses have recently shown that in addition to their endothelial characteristics, iBMECs also express several epithelial markers [31] and may not have a purely endothelial cell identity [76]. Comparison of published RNA-seq data from iBMECs, immortalized BMEC cell lines, and immunopanned in vivo brain endothelial cells (Fig. 3a) highlights the differences between iPSC-derived and in vivo-sourced brain endothelium and emphasizes the need for further improvements in differentiation methods. Interestingly, in vivo-sourced brain endothelium shows enrichment of gene ontology terms related to interferon signaling and immune response (Fig. 3b). This is likely attributable to the immortalization procedure [77] or isolation by immunopanning and indicates the need for a better in vivo, artifact-free transcriptomic analysis of adult human BMECs in order to benchmark iPSC-derived BMECs. Conversely, iBMECs are enriched for pathways associated with cell proliferation, patterning, and extracellular matrix interaction (Fig. 3c) which may be reflective of the cells being at an earlier developmental stage.

Despite iBMECs expressing many of the necessary solute channel and ATP-binding cassette transporters [18, 38, 39], expression levels of some of these transporters fall below in vivo values [31, 72, 78], suggesting that further maturation of iBMECs or modification to culturing conditions may be required to match in vivo levels. The current explosion of single-cell RNA-sequencing will likely help delineate some of the in vitro versus in vivo differences and has recently uncovered the vast and previously underappreciated heterogeneity in brain microvasculature [79], which has not been addressed in iPSC-derived BBB models. Regional differences especially between gray and white matter vasculature have also been reviewed recently [80] and will need to be taken into consideration in future studies using BBB models. Organ-chip technology has expanded our ability to more closely replicate the in vivo microenvironment, but the small diameters, shear stress forces, and complex vascular networks observed in brain capillaries are difficult to recreate with current platforms. However, this is a quickly evolving field that is already beginning to address some of these challenges.

With the increased use of iBMECs and their potential future applicability for preclinical studies, the need for validation of such models has become paramount. DeStefano and colleagues [81] recently outlined a set of 12 criteria for benchmarking and validating in vitro BBB models. These include critical assessments of permeability, the ultrastructure of tight junctions, expression of BBB markers, and transporter function. However, the limited availability of in vivo human permeability data has made it difficult to verify in vitro iPSC-derived BBB permeability measurements for many drugs, but assessing permeability of compounds such as PET radioligands with known in vivo human values now provides further validation of models [74]. Other assays that demonstrate functional characteristics of endothelial cells have also been developed and can be applied to iPSC-derived BBB models, for example, uptake of fluorescently-labeled low-density lipoprotein and endothelial cell tube formation in Matrigel [18]. Additional obstacles in modeling drug permeability, such as variable expression of drug transporters as well as drug absorption by cell culture plastic [82] and PDMS [83] used in many BBB models, are beginning to be addressed with improvements in differentiation protocols and advances in cell culture materials, respectively. Additionally, as new methods to generate iBMECs continue to evolve, the reproducibility of differentiation protocols needs to be considered. iBMEC differentiation efficiency and barrier formation have been shown to vary based on cell line [12], cell seeding density [16], reagent source [84], and response to media components [23, 62]. Developing robust protocols that are less sensitive to these variables will undoubtedly improve intra- and inter-lab reproducibility.

Despite these challenges, the human iPSC-derived BBB models discussed in this review are shown to mirror several known in vivo drug permeabilities and model various aspects of disease and microbial infection. These models also have many functional properties that mimic the in vivo BBB and can be combined with other cells of the NVU for personalized and predictive in vitro modeling. The coming years will likely see continued development of iPSC-derived BBB models leading to improvements in our understanding of human BBB function, new insights into mechanisms of neurological disease, and the development of novel BBB-permeable drugs to target the CNS.

## Data Availability

The datasets analyzed during the current study were all obtained from previous publications and are publicly available in the Gene Expression Omnibus (GEO) repository under Accession numbers: GSE76531, GSE108012, GSE97100, GSE97575, GSE97324, GSE129290, and GSE73721.

## Abbreviations

BBB: Blood–brain barrier
iPSC: Induced pluripotent stem cell
BMEC: Brain microvascular endothelial cell
iBMECs: iPSC-derived BMECs
NVU: Neurovascular unit
CNS: Central nervous system
TEER: Transendothelial electrical resistance
RA: Retinoic acid
ETA: Efflux transporter activity
PDMS: Polydimethylsiloxane
AD: Alzheimer’s disease
ALS: Amyotrophic lateral sclerosis
HD: Huntington’s disease
PD: Parkinson’s disease
Aβ: β-amyloid
TNFα: Tumor necrosis factor alpha
TH: Thyroid hormone
PET: Positron emission tomography
GO: Gene ontology
PCA: Principal component analysis
BEC: Brain endothelial cell
